# A Novel Stripe Noise Removal Model for Infrared Images

**DOI:** 10.3390/s22082971

**Published:** 2022-04-13

**Authors:** Mingxuan Li, Shenkai Nong, Ting Nie, Chengshan Han, Liang Huang, Lixin Qu

**Affiliations:** 1 Changchun Institute of Optics, Fine Mechanics and Physics, Chinese Academy of Sciences, Changchun 130033, China; limingxuan17@mails.ucas.ac.cn (M.L.); nongshenkai19@mails.ucas.ac.cn (S.N.); nieting@ciomp.ac.cn (T.N.); hanchengshan@ciomp.ac.cn (C.H.); qulixin@ciomp.ac.cn (L.Q.); 2 University of Chinese Academy of Sciences, Beijing 100049, China

**Keywords:** infrared images, stripe noises, adaptive edge-preserving operator (AEPO), alternating direction method of multipliers (ADMM)

## Abstract

Infrared images often carry obvious streak noises due to the non-uniformity of the infrared detector and the readout circuit. These streak noises greatly affect the image quality, adding difficulty to subsequent image processing. Compared with current elimination algorithms for infrared stripe noises, our approach fully utilizes the difference between the stripe noise components and the actual information components, takes the gradient sparsity along the stripe direction and the global sparsity of the stripe noises as regular terms, and treats the sparsity of the components across the stripe direction as a fidelity term. On this basis, an adaptive edge-preserving operator (AEPO) based on edge contrast was proposed to protect the image edge and, thus, prevent the loss of edge details. The final solution was obtained by the alternating direction method of multipliers (ADMM). To verify the effectiveness of our approach, many real experiments were carried out to compare it with state-of-the-art methods in two aspects: subjective judgment and objective indices. Experimental results demonstrate the superiority of our approach.

## 1. Introduction

The readout circuit of an infrared detector is highly inconsistent. The non-uniformity of the detector is often manifested in the image as streak noises, which directly affect the imaging quality, and can even hinder subsequent image processing [1,2,3], namely, image classification, target detection, and target recognition. Therefore, it is important to explore how to remove stripe noises while preserving image details. This paper aims to separate stripe noise components from a target infrared image and obtain the information components that preserve image details.

In recent years, many scholars have been devoted to the removal of stripe noises from images, and have proposed various methods such as frequency domain filtering, wavelet transform, statistical matching, and total variation.

In 1987, Quarmby adopted a spatial frequency domain filter to remove stripe noises, according to the frequency difference between stripe noises and the target information in the frequency domain [4]. Soon, wavelet transform was introduced to eliminate stripe noises [5,6,7]. As an example of this, a contrastive method is multi-scale guided filter (MSGF) [8]. Through wavelet transform, the target image is divided into high- and low-frequency components before removing stripe noises, which is possible due to the ability of wavelet transform to describe the local frequency components of signals. However, there are two limitations to the frequency-domain filtering of stripe noises: First, this strategy cannot easily differentiate between stripe noises and the target information, unless the stripe noises are highly regular. Second, this strategy may remove edge and texture information when the target information is relatively complex. As a result, the original information may be lost, and artifacts may even appear.

Another popular approach to stripe noise removal is statistical matching, which is often used in engineering. Originally applied to the marine data of MOS-B, this approach assumes that the response of each pixel on the sea surface should output equal electrical levels [9]. Under this assumption, the output features of each pixel are obtained, including the gain and bias coefficients. Then, the denoised target image is obtained through correction based on these parameters. Later, some scholars combined local constant statistical constraint with neural networks [10], or wavelet transform coupled with gradient equalization (WAGE) [11], to remove stripe noises. The former approach views each row of pixels as having the same standard deviation and mean, and takes these values as the median of local channel statistics, thereby correcting the stripe noises caused by image non-uniformity. The latter approach concentrates the stripe noise components in each vertical component of wavelet transform, and removes the stripe noises through column equalization. However, this approach, failing to consider strong edge information, cannot effectively remove noises, when the stripe noises if they are unevenly distributed.

Recently, a class of popular methods emerged based on total variation minimization. The earliest method in this class was proposed by Antonin Chambolle in 2004. These methods construct a cost function to derive the minimization equation, which represents the ideal image features, and solves the ideal image that minimizes the equation through gradient descent [12]. Then, some scholars adopted the L1-norm of the difference between the original image and the denoised image as the cost function [13] to optimize denoising. Some scholars combined the above three methods to remove noises and proposed total variation coupled with a guided filter (TVGF) for stripe noises. Firstly, frequency domain filtering was employed to extract the high-frequency stripe noise components. Secondly, the total variation model was adopted to implement gradient equalization and eliminate strong interference noises. Finally, the smooth image was used as a guide to eliminate stripe noises [14].

Most existing stripe noise removal methods for infrared images focus on the denoising degree of the image, but rarely consider the structural features of the stripe noises, not to mention the differentiation between image edges and stripe noises. Hence, many current stripe noise removal algorithms for infrared images either have insufficient denoising ability (the denoised image still has residual noises), or have excessive denoising ability (the denoised image loses information). In previous studies, stripe noises in infrared images were regarded as fixed pattern noises, i.e., additive noises. In other words, the original image was assumed to contain two types of components: noise and information [15,16,17]. The denoising problem can be regarded as the extraction of noise components from the original image, that is, the estimation of noise components. Considering the above problems, this paper tries to combine prior information of infrared stripe noises with edge extraction, in order to improve the denoising effect while preserving the edge information.

In this paper, the L1-norm, which is often used as an error function, is adopted to represent the stripe noise features of the infrared images. Owing to their stripe property, the noise components clearly differ from information components in terms of structure and direction. After exploring the directionality and cross-directionality of the noise components, this paper presents a new optimization-based stripe noise removal model for infrared images, capable of adaptively preserving the edges. In addition, the alternating direction method of multipliers (ADMM) is utilized to solve the model [18,19]. The proposed method has the following strengths:

1. This paper constructs a convex optimization model, drawing on the unique directional, cross-directional, and structural properties of stripe noises. The model makes full use of the prior information on stripe noise classes and actual information components to improve the noise separation.

2. Based on the global sparsity and gradient sparsity of stripe noises, this paper adopts the L1-norm to constrain the overall sparsity of stripe noise and the gradient sparsity along the stripe direction, producing a nonconvex optimization model. This facilitates the solution of the optimal results.

3. This paper proposes an adaptive edge-preserving operator (AEPO), which ensures that the edges will not be over-smoothed or distorted during the optimization process.

## 2. Preliminaries

Stripe noises differ from other noises in directionality and structure. According to these unique properties, this section focuses on designing the regular terms to remove the noises of infrared images.

### 2.1. Stripe Noise Model

In the field of infrared image denoising, stripe noises are widely regarded as additive noises with strong structuredness and directionality. The optimization-based denoising approaches emphasize the design of proper regular terms, in the light of the properties of stripe noises or the target image.

**Figure 1:**
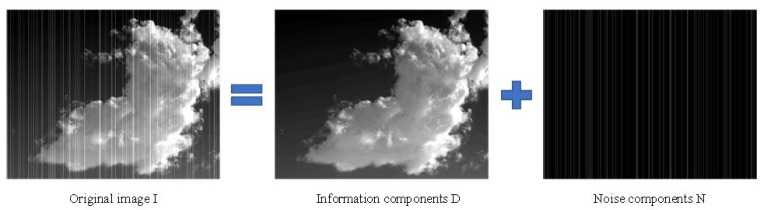
Block diagram of the proposed approach.

The first step is to mathematically depict the correlations of the original image with the stripe noise components and information components:

(1) I(i,j)=D(i,j)+N(i,j)

where I(i,j),D(i,j), and N(i,j) are the original image outputted by the infrared detector, [d=Li.] the information components of the noise-free imagethe information components after the removal of stripe noises, and the stripe noise components at pixel (i,j), respectively. Since our strategy considers the entire image, Formula (1) can be rewritten as a matrix:

(2) I=D+N

where, I, D, and N are the discrete vectors of I(i,j),D(i,j), and N(i,j), respectively. Starting with the properties of stripe noises N, this paper presents a denoising approach for stripe noises based on optimization. The proposed approach can remove stripe noises from the original image and preserve the effective information in the image as much as possible. Figure 1 illustrates the procedure of the approach.

### 2.2. Properties of Stripe Noises

**Figure 2:**
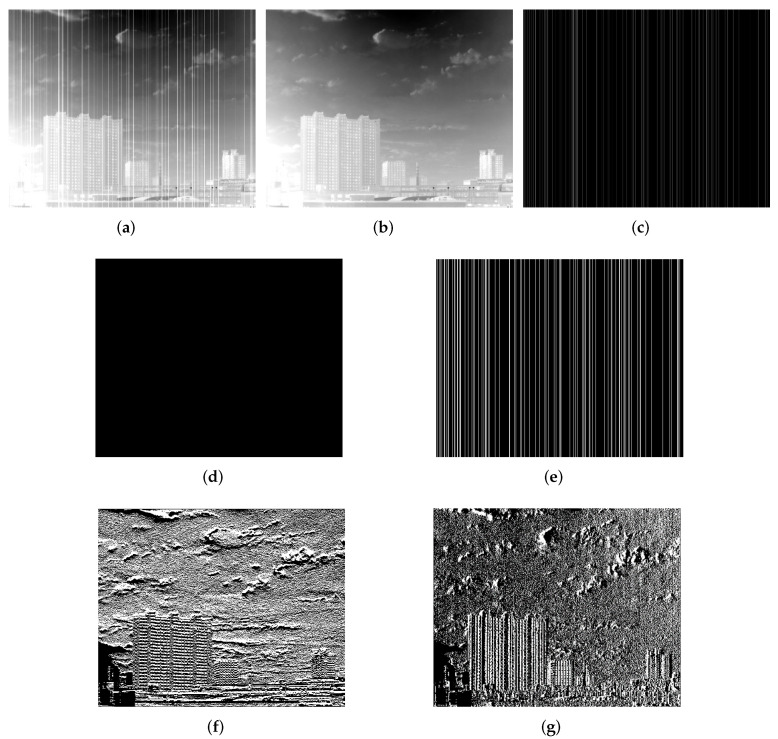
Difference between information components and stripe noises: (**a**) Original image; (**b**) Information components; (**c**) Noise components; (**d**) Vertical gradient of noise components; (**e**) Horizontal gradient of noise components; (**f**) Vertical gradient of information components; (**g**) Horizontal gradient of information components.

**Figure 3:**
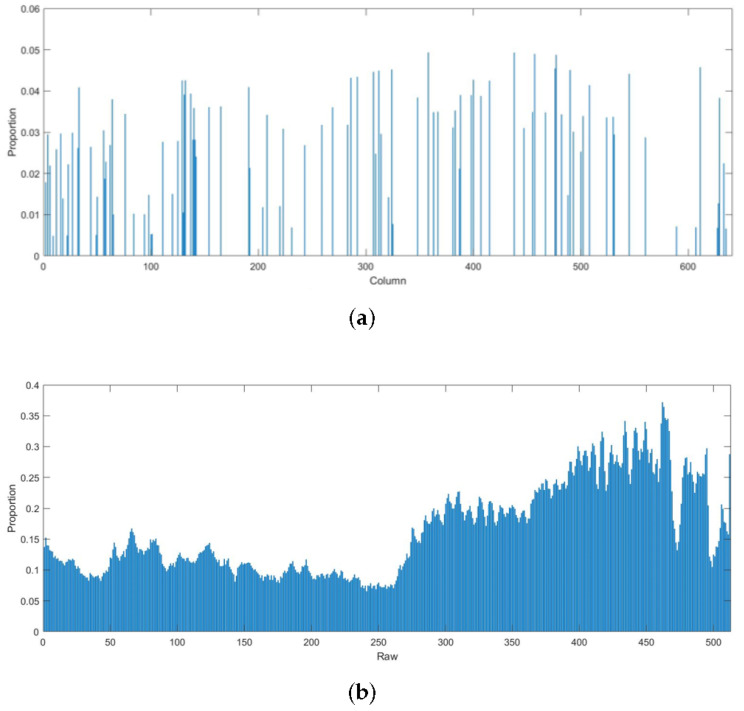
Difference between proportion of the L1-norm for information components and stripe noises: (**a**) Proportion of the L1-norm for each column of noise components in the L1-norm for the vertical gradient of the original image; (**b**) Proportion of the L1-norm for each row of information components in the L1-norm for the horizontal gradient of the original image.

To improve the denoising of stripes noises in infrared images, it is essential to fully understand all prior knowledge of the topic, and to constrain the features of these noises with the corresponding regular terms. Figure 2 and Figure 3 show some properties of stripe noises. It can be seen that the stripe noise components are more directional and structurally regular than the information components in the original image.

(1) Directionality

Figure 2 shows the differences in properties between stripe noise components and information components in the original image. Comparing Figure 2d with Figure 2f, it is clear that the vertical gradient of stripe noise components is much smoother than that of information components. Comparing Figure 2e with Figure 2g, it is apparent that the horizontal gradient of stripe noise components is not as smooth as that of information components. In addition, the gradient domain of stripe noises is sparse in the vertical direction. To distinguish between stripe noise components and information components, the sparsity needs to be constrained into the vertical gradient field. The L0-norm is the best tool to describe sparsity [20,21]. Hence, the following regular term formula can be established as:

(3) $$P_{1}\left(N\right)={∥d_{y}N∥}_{0}$$

where $d_{y}$ is the convolutional gradient operator in the vertical direction. Since the L0-norm is not nonconvex, this term was sparsely represented by the nonconvex L1-norm instead of the L0-norm [22,23]. Therefore, we denote this regular term as:

(4) $$P_{1}\left(N\right)={∥d_{y}N∥}_{1}$$

This kind of optimization-based approach often uses the root-mean square error (RMSE) or square error between the original image and noise components as the fidelity term [24], such that the image will not be distorted due to excessive denoising. The fidelity term can be expressed as:

(5) $$P_{2}\left(N\right)={∥I-N∥}_{2}^{2}$$

Or:

(6) $$P_{2}\left(N\right)={\left(I^{2}-N^{2}\right)}^{1/2}$$

I–N are essentially information components D. Neither of the above fidelity terms take the properties of information components D or noise components N into account. As shown in Figure 2, there are obvious stripes in the horizontal gradient domains of the original image and the stripe noise components. Meanwhile, the horizontal gradient domain of the information components is relatively smooth. Thus, this is adopted as the fidelity term. In other words, the horizontal gradient of the information components was depicted as the L1-norm of the horizontal gradient difference between the original image and the noise components:

(7) $$P_{2}\left(N\right)={∥d_{x}I-d_{x}N∥}_{1}$$

where $d_{x}$ is the convolutional gradient operator in the horizontal direction. In addition, Figure 3b shows that the L1-norm for each row of information components in the L1-norm takes up a small proportion of the horizontal gradient of the original image. Therefore, this regular term can ensure smoothness in the horizontal direction. However, the vertical edges could be over-smoothed during the optimization, causing the loss of edge information. To prevent this problem, the image edges were recognized in advance, edge pixels were assigned a small weight operator, and non-edge pixels were assigned a large weight operator. Then, Formula (7) can be modified as:

(8) $$P_{2}\left(N\right)=\Omega_{edge}{∥d_{x}I-d_{x}N∥}_{1}$$

where $\Omega_{\mathrm{edge}\;}$ assigns different weights to the edge pixels in the original image:

(9) $$\Omega_{\mathrm{edge}\;}\left(i,j\right)=\left\{\begin{array}{c} \alpha ,\;\mathrm{if}\;(i,j)\;\mathrm{is}\;\mathrm{edge}\;\mathrm{pixel}\;\\ 1,\;\mathrm{else}\; \end{array}\right.$$

where α is a constant. Additionally, the method for obtaining the value of α is introduced in Formula (34).

(2) Structuredness

As shown in Figure 2c, the stripe noises in the infrared image exist in columns. The pixels in the stripe-free areas equal zero. Hence, stripe noise components can be regarded as a sparse matrix, represented by the L0-norm:

(10) $$P_{3}\left(N\right)={∥N∥}_{0}$$

Similar to the sparse matrix in the vertical gradient domain described in Section 2.1, the L0-norm is not nonconvex. Thus, the regular term can be expressed by the nonconvex L1-norm:

(11) $$P_{3}\left(N\right)={∥N∥}_{1}$$

As shown in Figure 3a, the L1-norm for each column of noise components takes up a very small proportion in the L1-norm for the vertical gradient of the original image, and ia able to realize the sparsity constraint.

### 2.3. AEPO Experiment

Using Formula (8) in Section 2.2, the fidelity term of the information components was obtained by examining the sparsity of the gradient domain for the actual information components of the target infrared image, which is perpendicular to the stripe direction. However, this fidelity term actually realizes the constraining effect through a smoothing operation vertically to the stripe direction. During optimization, the edge pixels are easily over-smoothed perpendicular to the stripe direction, causing loss of information. To overcome the problem, this paper presents an AEPO based on edge contrast. The operator aims to adaptively adjust the weights of edge pixels through the optimization process, and aims to prevent the information loss induced by over-smoothing of these pixels.

Experiments were conducted to summarize the relationship between the value of the AEPO and the contrast of edge pixels. The denoising effect of the AEPO at different edge contrasts was measured by structural similarity (SSIM), which is a full-reference evaluation index [25,26].

**Figure 4:**
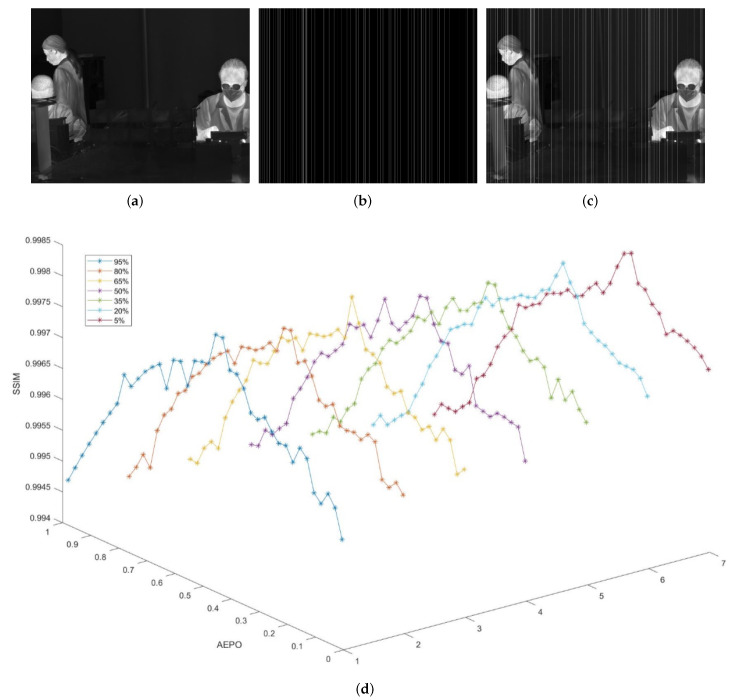
Experiments on the AEPO: (**a**) reference image; (**b**) noisy image; (**c**) test image; (**d**) correlation of SSIM with AEPO and edge contrast.

Figure 4 shows the details of these experiments. Figure 4a presents a reference image without any stripe noise; Figure 4b provides an image containing random stripe noises; Figure 4c displays the test image that combines the reference image with the noises. To fully verify the relationship between the AEPO value and edge contrast, 30 edge pixels were selected as objects from the original image, and the mean SSIM of these pixels was solved after manually adjusting the edge contrast. On this basis, the authors discussed how to optimize the AEPO. In Figure 4d, the different colored curves reflect the influence of the AEPO value on the SSIM at different contrasts of edge pixels. The experimental results clearly demonstrate that when the edge contrast remained constant, there was an optimal value of the AEPO leading to the best denoising effect of the edge pixels. With the decline in edge contrast, the value of the optimal AEPO decreased.

## 3. Methodology

Based on the properties of stripe noises and regular terms identified in the preceding section, this section finalizes the stripe noise removal model for infrared images, and details how to solve the model through the ADMM. During the separation of stripe noises, our model obtains an AEPO based on the edge contrast. In this way, the noise components are extracted without sacrificing edge information.

### 3.1. Model

The above analysis reveals large differences between the noise components and the information components of infrared images in terms of structure and directionality. The three terms $P_{1}\left(N\right),P_{2}\left(N\right)$, and $P_{3}\left(N\right)$ can be combined to obtain the final stripe noise optimization model:

(12) $$N=\arg\min_{N}\left\{\lambda_{1}{∥d_{y}N∥}_{1}+\lambda_{2}{∥N∥}_{1}+\lambda_{3}\Omega_{edge}{∥d_{x}I-d_{x}N∥}_{1}\right\}$$

where, $\lambda_{1},\lambda_{2}$, and $\lambda_{3}$ are used to balance the different regular terms. Firstly, the stripe noise components N that minimize Formula (12) are solved. Then, the denoised information components can be estimated through the transform below:

(13) D=I−N

### 3.2. ADMM Optimization

Finding the second derivative is the most direct way to optimize the convergence matrix above. Nevertheless, the regular terms of the regularization model (12), which is based on the L1-norm, are not continuously differentiable, making derivation difficult. As a popular machine learning tool, the ADMM provides an effective solution to the regular terms of the L1-norm. In essence, the algorithm optimizes the parts for unconstrained optimization through block coordinate (12). The specific solving process is explained below.

For the three regular terms, three auxiliary variables are introduced to substitute these regular terms, namely, G=$d_{y}N,\;T=N$, and $U=d_{x}I-d_{x}N$. Then, the minimization of Formula (12) is equivalent to:

(14) $$\begin{array}{c} \arg\min_{N,G,U}\left\{\lambda_{1}{∥G∥}_{1}+\lambda_{2}{∥T∥}_{1}+\lambda_{3}\Omega_{\mathrm{edge}\;}{∥U∥}_{1}\right\}\\ \;s.t.\;G=d_{y}N,\;T=N,\;U=d_{x}I-d_{x}N \end{array}$$

The convex optimization problem (14) may be further converted into an augmented Lagrangian function:

(15) $$\begin{array}{cc} \; & \arg\min_{N,G,U}\lambda_{1}{∥G∥}_{1}+\lambda_{2}{∥T∥}_{1}+\lambda_{3}W_{\mathrm{edge}\;}{∥U∥}_{1}+m_{1}^{T}\left(d_{y}N-G\right)+m_{2}^{T}\left(N-T\right)+\\ \; & m_{3}^{T}\left(d_{x}I-d_{x}N-U\right)+\frac{\rho_{1}}{2}{∥d_{y}N-G∥}_{2}^{2}+\frac{\rho_{2}}{2}{∥N-T∥}_{2}^{2}+\frac{\rho_{3}}{2}{∥d_{x}I-d_{x}N-U∥}_{2}^{2} \end{array}$$

where, $m_{1},m_{2}$, and $m_{3}$ are the Lagrange multipliers of the three constraints, respectively; $\rho_{1},\rho_{2}$, and $\rho_{3}$ are three penalties. Then, Formula (15) can be converted into four sub-items for iterative solution:

a. G problem

(16) $$G=\arg\min_{G}\lambda_{1}{∥G∥}_{1}+m_{1}^{T}\left(d_{y}N-G\right)+\frac{\rho_{1}}{2}{∥d_{y}N-G∥}_{2}^{2}$$

According to Formula (12) in Reference [27], for solving for X such that the following formula is minimized:

(17) $$\arg\min_{X}{∥X-B∥}_{2}^{2}+2\lambda {∥X∥}_{1}$$

It can be directly obtained that:

(18) X=soft(B,λ)=sign(B)max(|B|−λ,0)

Thus, Formula (16) can be converted into:

(19) $$G=\arg\min_{G}\lambda_{1}{∥G∥}_{1}+\frac{\rho_{1}}{2}{∥d_{y}N-G+\frac{m_{1}}{\rho_{1}}∥}_{2}^{2}$$

Following the solution principle of Formula (17), it can be solved by:

(20) $$G^{k+1}=\mathrm{soft}\left(d_{y}N^{k}+\frac{m_{1}^{k}}{\rho_{1}},\frac{\lambda_{1}}{\rho_{1}}\right)$$

where, *k* is the number of iterations.

b. T problem

(21) $$T=\arg\min_{T}\lambda_{2}{∥T∥}_{1}+m_{2}^{T}\left(N-T\right)+\frac{\rho_{2}}{2}{∥N-T∥}_{2}^{2}$$

Similar to the G problem, it can be solved that:

(22) $$T^{k+1}=\mathrm{soft}\left(N^{k}+\frac{m_{2}^{k}}{\rho_{2}},\frac{\lambda_{2}}{\rho_{2}}\right)$$

c. U problem

(23) $$U=\arg\min_{U}\lambda_{3}\Omega_{edge}{∥U∥}_{1}+m_{3}^{T}\left(d_{x}I-d_{x}N-U\right)+\frac{\rho_{3}}{2}{∥d_{x}I-d_{x}N-U∥}_{2}^{2}$$

It can be solved that:

(24) $$U^{k+1}=\mathrm{soft}\left(d_{x}I-d_{x}N^{k}+\frac{m_{3}^{k}}{\rho_{3}},\frac{\lambda_{3}\Omega_{edge}}{\rho_{3}}\right)$$

d. N problem:

(25) $$\begin{array}{cc} \; & N=\arg\min_{N}m_{1}^{T}\left(d_{y}N-G\right)+m_{2}^{T}\left(N-T\right)+m_{3}^{T}\left(d_{x}I-d_{x}N-U\right)+\frac{\rho_{1}}{2}∥d_{y}N-\\ \; & G∥{}_{2}^{2}+\frac{\rho_{2}}{2}∥N-T∥{}_{2}^{2}+\frac{\rho_{3}}{2}∥d_{x}I-d_{x}{N-U∥}_{2}^{2} \end{array}$$

Formula (25) can be simplified as:

(26) $$\begin{array}{cc} \; & N=\arg\min_{N}\left\{\frac{\rho_{1}}{2}{∥d_{y}N-G+\frac{m_{1}}{\rho_{1}}∥}_{2}^{2}+\frac{\rho_{2}}{2}{∥N-T+\frac{m_{2}}{\rho_{2}}∥}_{2}^{2}+\frac{\rho_{3}}{2}∥d_{x}I-d_{x}N-U+\right.\\ \; & \left.\frac{m_{3}}{\rho_{3}}{∥}_{2}^{2}\right\} \end{array}$$

This is a quadratic optimization with differentiability. It is equivalent to solving the following linear system. Through the direct derivation of Formula (26):

(27) $$\begin{array}{c} \rho_{1}\;d_{y}{}^{T}⊗d_{y}⊗N^{k+1}+\rho_{2}N^{k+1}+\rho_{3}\;d_{x}{}^{T}⊗d_{x}⊗N^{k+1}=\rho_{1}\;d_{y}{}^{T}⊗\left(G^{k+1}-\frac{m_{1}}{\rho_{1}}\right)+\\ \rho_{2}\left(T^{k+1}-\frac{m_{2}}{\rho_{2}}\right)+\rho_{3}\;d_{x}{}^{T}⊗\left(d_{x}I-U^{k+1}+\frac{m_{3}}{\rho_{3}}\right) \end{array}$$

where ⊗ denotes convolution. It is very difficult to solve a formula involving convolution. This paper introduces the Fourier transform to convert the convolution in the time domain into multiplication in the frequency domain:

(28) $$\begin{array}{c} \left[\rho_{1}F\left(d_{y}^{T}\right)*F\left(d_{y}\right)+\rho_{2}+\rho_{3}F\left(d_{x}{}^{T}\right)*F\left(d_{x}\right)\right]*F\left(N^{k+1}\right)=\rho_{1}F\left(d_{y}^{T}\right).*\\ F\left(G^{k+1}-\frac{m_{1}}{\rho_{1}}\right)+\rho_{2}F\left(T^{k+1}-\frac{m_{2}}{\rho_{2}}\right)+\rho_{3}F\left(d_{x}^{T}\right).*F\left(d_{x}I-U^{k+1}+\frac{m_{3}}{\rho_{3}}\right) \end{array}$$

By left division of the matrix, we obtain:

(29) $$\begin{array}{c} F\left(N^{k+1}\right)=\left[\rho_{1}F\left(d_{y}^{T}\right).*F\left(G^{k+1}-\frac{m_{1}}{\rho_{1}}\right)+\rho_{2}F\left(T^{k+1}-\frac{m_{2}}{\rho_{2}}\right)+\rho_{3}F\left(d_{x}^{T}\right).*\right.\\ \left.F\left(d_{x}I-U^{k+1}+\frac{m_{3}}{\rho_{3}}\right)\right]./\left[\rho_{1}F\left(d_{y}^{T}\right).*F\left(d_{y}\right)+\rho_{2}+\rho_{3}F\left(d_{x}^{T}\right).*F\left(d_{x}\right)\right] \end{array}$$

Then, the inverse Fourier transform of Formula (29) was implemented to obtain the expression for stripe noises N:

(30) $$\begin{array}{c} N^{k+1}=F^{-1}\left\{\left[\rho_{1}F\left(d_{y}^{T}\right)*F\left(G^{k+1}-\frac{m_{1}}{\rho_{1}}\right)+\rho_{2}F\left(T^{k+1}-\frac{m_{2}}{\rho_{2}}\right)+\rho_{3}F\left(d_{x}^{T}\right).*\right.\right.\\ \left.\left.F\left(d_{x}I-U^{k+1}+\frac{m_{3}}{\rho_{3}}\right)\right]./\left[\rho_{1}F\left(d_{y}^{T}\right)*F\left(d_{y}\right)+\rho_{2}+\rho_{3}F\left(d_{x}^{T}\right).*F\left(d_{x}\right)\right]\right\} \end{array}$$

where .* is the point multiplication of two matrices; ./ is the point division of two matrices; F() is the Fourier transform; $F^{-\infty }\left(\right)$ is the inverse Fourier transform. Note that the complete convolution of a matrix will change its size. It is highly necessary to conduct normalization during the computing. After each iteration, the Lagrange multipliers must be updated by [28]:

(31) $$\left\{\begin{array}{c} m_{1}^{k+1}=m_{1}^{k}+\rho_{1}\left(d_{y}N^{k+1}-G^{k+1}\right)\\ m_{2}^{k+1}=m_{2}^{k}+\rho_{2}\left(N^{k+1}-T^{k+1}\right)\\ m_{3}^{k+1}=m_{3}^{k}+\rho_{3}\left(d_{x}I-d_{x}N^{k+1}-U^{k+1}\right) \end{array}\right.$$

Finally, the noise components $N^{k+1}$ of the original image were obtained, and the final denoised image was derived through $D=I-N^{k+1}$.

### 3.3. AEPO

According to the experiments in Section 2.3, we learn that the selection of the AEPO has a large impact on the effectiveness of the algorithm for stripe noise removal. Figure 4a–d show that, to optimize the denoising effect, the AEPO value must increase with the edge contrast.

To quantify the contrast of edge pixels, a formula was defined for the normalized edge contrast:

(32) $$C_{edge}\left(i,j\right)=\left(|I\left(i,j\right)-E\left(NBH\left(i,j\right)\right)|/2^{n}\right)*100\%$$

where, $C_{\mathrm{edge}\;}\left(i,j\right)$ is the edge contrast of the pixel in row i and column j of the image; NBH(i,j) is the neighboring pixel of pixel (i,j) in the direction perpendicular to stripe noises; E() is the averaging operation; *n* is the digits of the image. Additionally, we can obtain the E(NBH(i,j)) as the following formula:

(33) E(NBH(i,j))=[I(i,j+1)+I(i,j−1)]/2

Through the above experiments, the AEPO at different edge contrasts can be optimized by:

(34) $$\Omega_{edge}\left(i,j\right)=\frac{\beta *e^{c_{edge}}}{e-1}+\theta$$

where β and θ are normalized parameters and *e* is the natural logarithm.

## 4. Experimental Results

In our experiments, our approach was compared with three state-of-the-art methods on three different image datasets. The contrastive methods are multi-scale guided filter (MSGF) for stripe noises [7], wavelet transform coupled with gradient equalization (WAGE) for stripe noises [11], and total variation coupled with guided filter (TVGF) for stripe noises [14]. To further demonstrate the effectiveness of the proposed method, an ablation experiment, that is, the non-AEPO method, was added to the comparison experiments. All the image data were shot using a LUSTER TB640-CL refrigerated medium wavefront infrared camera at the resolution of 640 × 512. All the experiments were run on MATLAB (R2020b), using a computer with 8 GB RAM and an AMD Ryzen 7 2700X Eight-Core Processor@3.70 GHz.

The experimental data were evaluated both subjectively and objectively. The subjective evaluation targets the edge details and denoising degree of the denoised image. For the experiments on real data, since there is no true image to use as a reference, we select the no-reference evaluation metrics of noise reduction (NR) [29,30], mean relative deviation (MRD) [30,31], and image distortion (ID) [32,33]. The definition of NR is shown in Formula (35), which reflects the overall performance of the denoised image. The definition of MRD is shown in Formula (36), which mirrors the ability to preserve image information in stripe-free areas. The definition of ID is shown in Formula (37), which demonstrates the degree of distortion for the denoised image. The denoising effect is positively correlated with the NR and the ID, and negatively with the MRD.

(35) $$\left\{\begin{array}{c} NR=N_{0}/N_{1}\\ N=\sum_{i=0}^{k}\mathrm{mean}P\left(u_{i}\right) \end{array}\right.$$

where $N_{0}$ and $N_{1}$ stand for the value of *N* in the original and de-striped images, respectively. $u_{i}$ is the frequency component produced by stripes. *N* is the total power of stripes’ noise in the mean power spectrum.

(36) $$MRD=\frac{1}{MN}\sum_{i=1}^{MN}\frac{\left|z_{i}-g_{i}\right|}{g_{i}}\times 100\%$$

where $g_{i}$ and $z_{i}$ are the pixel values of point *i* in the original image and the image after stripe noise removal, respectively. Additionally, MN represents the number of all pixels in the selected area.

(37) $$\left\{\begin{array}{c} ID=S_{1}/S_{0}\\ S=\sum_{j\neq 1}^{N-1}\mathrm{mean}P\left(u_{j}\right) \end{array}\right.$$

where $S_{0}$ and $S_{1}$ stand for the value of *S* in original image and the de-striped image, respectively. $u_{j}$ is the frequency component caused by the raw image without stripes. *S* stands for the total power of the clean image in the mean power spectrum.

### 4.1. Parameter Analysis

**Figure 5:**
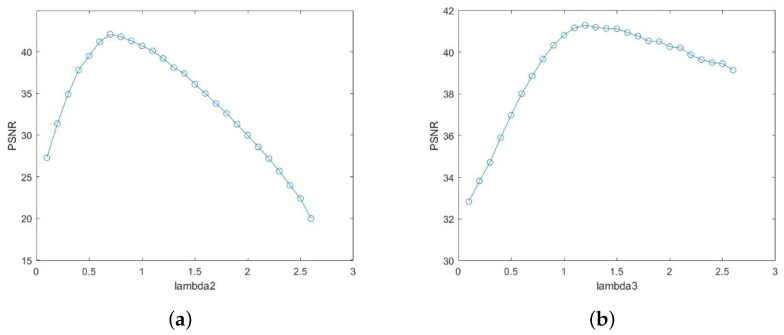
Influence of regular terms on the PSNR: (**a**) relationship between $\lambda_{2}$ and PSNR; (**b**) relationship between $\lambda_{3}$ and PSNR.

Taking Figure 4 as an example, the three regular terms were subjected to a sensitivity analysis, with the aim to verify the importance of the key parameters to our approach. For such reference experiments, the full-reference evaluation metric is used to better reflect the denoising performance of the algorithm. As a representative of the full-reference evaluation index, the peak signal-to-noise ratio (PSNR) is often widely used to determine the parameter because it can reflect the denoising effect of the image. Thus, we selected the PSNR metric to evaluate this experiment to prove the validity of the parameter selection. Firstly, $\lambda_{1}$ was empirically set to 1. Figure 5 shows the relationship between PSNR and regular terms $\lambda_{2}$ and $\lambda_{3}$ [34]. The results in Figure 5 prove that the selected $\lambda_{2}$ and $\lambda_{3}$ indeed affect the denoising performance. Similar results were obtained in other experiments. According to the experimental findings, it was determined that $\lambda_{1}=1$, $\lambda_{2}=0.7$ and $\lambda_{3}=1.2$. As for penalties, their values were empirically set to $\rho_{1}=\rho_{2}=\rho_{3}=0.15$. In Formula (12), the optimal values of β and θ fall in [0.15,0.20], and [0.4,0.5], respectively. In our experiments, the two parameters were empirically set to β=0.18 and θ=0.46, respectively.

### 4.2. Experimental Contents

**Figure 6:**
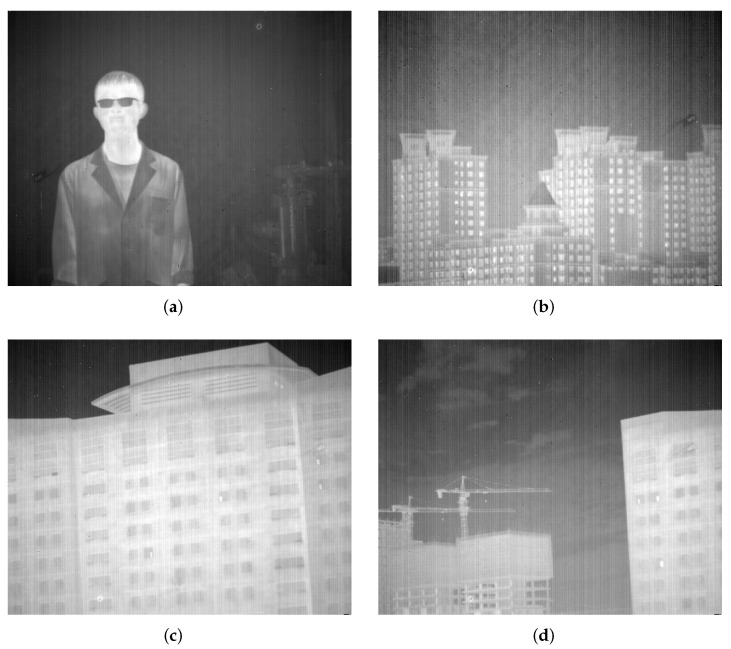
Experimental images: (**a**) a person; (**b**) buildings against the sky; (**c**) a single building; (**d**) complex buildings.

In order to prove the universality and effectiveness of the presented approach in this paper, four infrared images of different scenes are selected to be the experimental subjects in these experiments. The first image is shown in Figure 6a, which contains a person with a large gray difference from the background, and an object with vertical edges that has a small gray difference from the background. The second image is shown in Figure 6b, which contains several buildings against the sky with a small difference in grayscale values from the stripe noises. Additionally, there are a large number of small vertical features. The third image is shown in Figure 6c, which contains a single building with the presence of features that do not have a distinct vertical texture. The fourth image is shown in Figure 6d, which mainly consists of complex buildings. Apart from the normal buildings, clouds and micro-objects such as tower cranes can be observed in the original image. In these four images, there are stripe noises with different grayscale differences, tiny vertical edge features and various texture characteristics. The excellent results of our approach can be fully illustrated.

#### 4.2.1. Ablation Experiments

**Figure 7:**
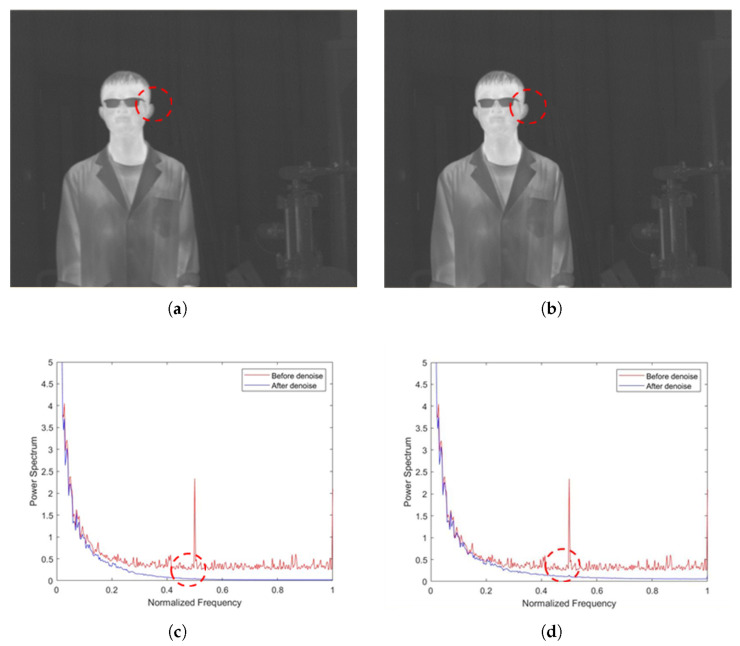
Denoising effects of ablation experiment on an image of a person: (**a**) non-AEPO; (**b**) our approach; (**c**) non-AEPO; (**d**) our approach.

The ablation experiments for our proposed method are shown in this section. As shown in Figure 7a,b, the non-AEPO method and our approach both display excellent denoising performance. However, Figure 7a shows that there is a significant blurring of the vertical edge characteristics (encircled by red dotted lines). Figure 7c,d compare the mean power spectral densities (MPSDs) of all rows in the images denoised by different methods with the MPSD of all rows in the original image, where the abscissa is the normalized frequency, and the ordinate is the MPSD of all rows. It is observed that the curve in Figure 7c is slightly smoother than that in Figure 7d (encircled by red dotted lines). This phenomenon verifies the difference between Figure 7a,b.

**Figure 8:**
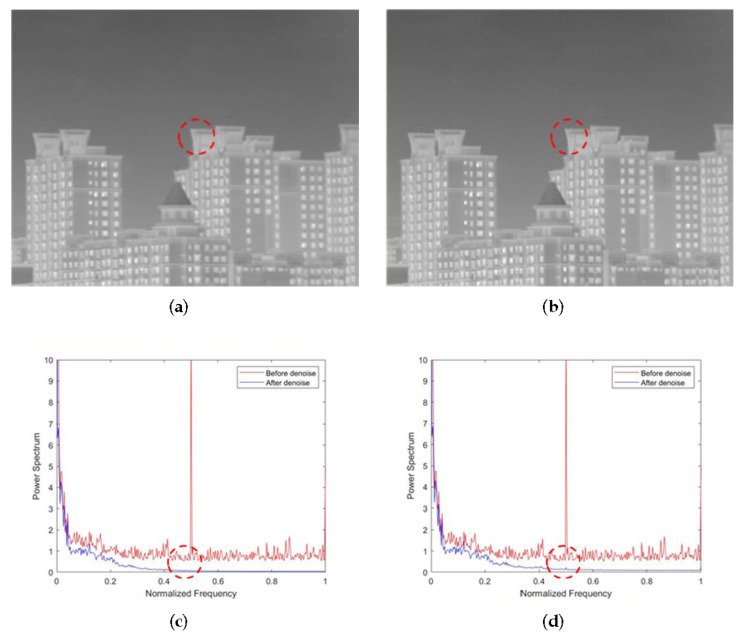
Denoising effects of ablation experiment on an image of buildings against the sky: (**a**) non-AEPO; (**b**) our approach; (**c**) non-AEPO; (**d**) our approach.

As shown in Figure 8a,b, the non-AEPO method and our approach both have good denoising performance. However, Figure 8a shows a significant blurring of the vertical edge characteristics on the buildings (encircled by red dotted lines). It is observed that the curve in Figure 8c is slightly smoother than that in Figure 8d (encircled by red dotted lines). This phenomenon verifies the difference between Figure 8a,d.

**Figure 9:**
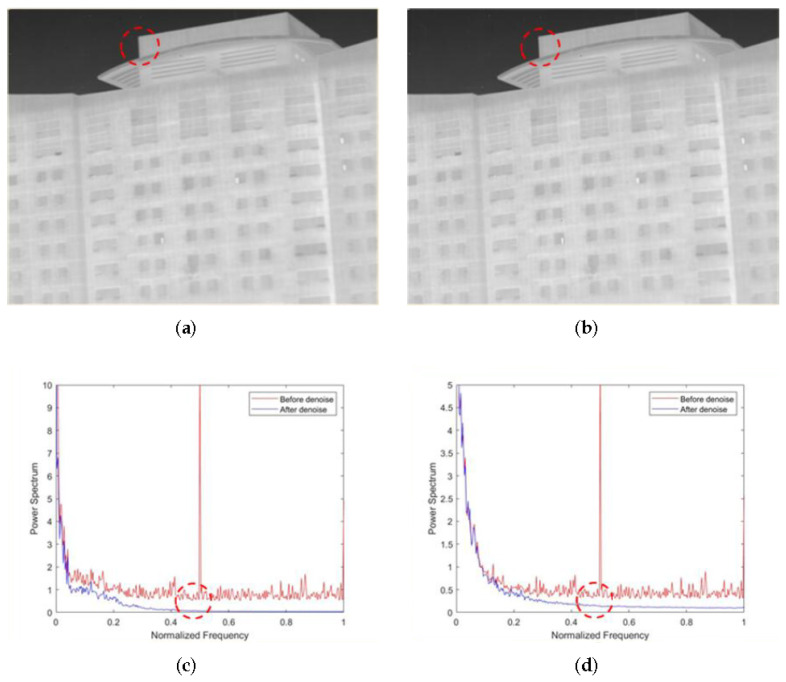
Denoising effects of ablation experiment on an image of a single building: (**a**) non-AEPO; (**b**) our approach; (**c**) non-AEPO; (**d**) our approach.

As shown in Figure 9a,b, the non-AEPO method and our approach both effectively remove the stripe noises from the original image. However, we can see in Figure 9a that there is significant blurring of the vertical edge characteristics on the top of the building (encircled by red dotted lines). It is observed that the curve in Figure 9c is slightly smoother than that in Figure 9d (encircled by red dotted lines). This phenomenon is consistent with the performance of the algorithms in Figure 9a,b.

**Figure 10:**
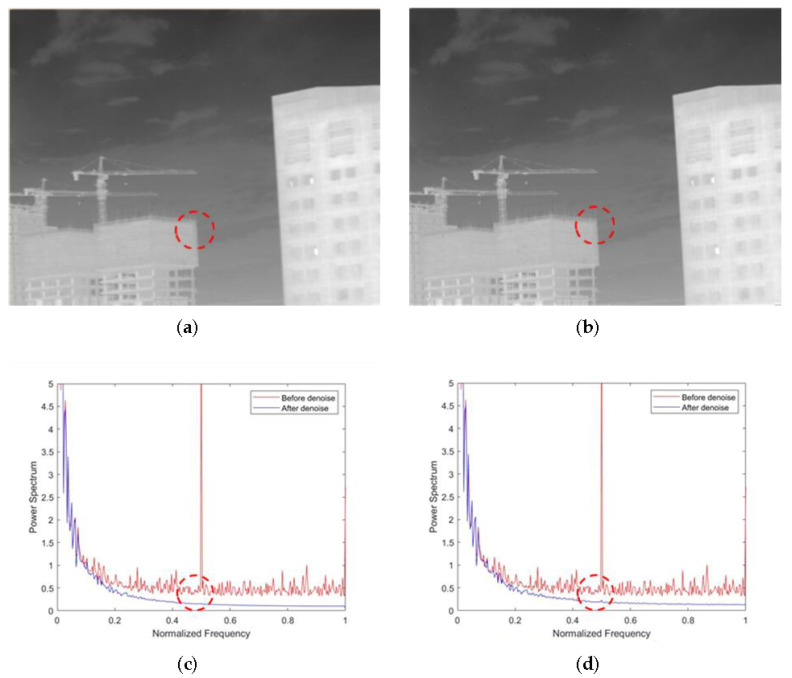
Denoising effects of ablation experiment on an image of complex buildings: (**a**) non-AEPO; (**b**) our approach; (**c**) non-AEPO; (**d**) our approach.

As shown in Figure 10a,b, the non-AEPO method and our approach both have excellent performance in removing stripe noise. However, we can see in Figure 10a that there is significant blurring of the vertical edge characteristics on the building (encircled by red dotted lines). It is observed that the curve in Figure 10c is slightly smoother than that in Figure 10d (encircled by red dotted lines). This is consistent with the phenomena reflected in Figure 10a,b.

#### 4.2.2. Comparison Experiments

**Figure 11:**
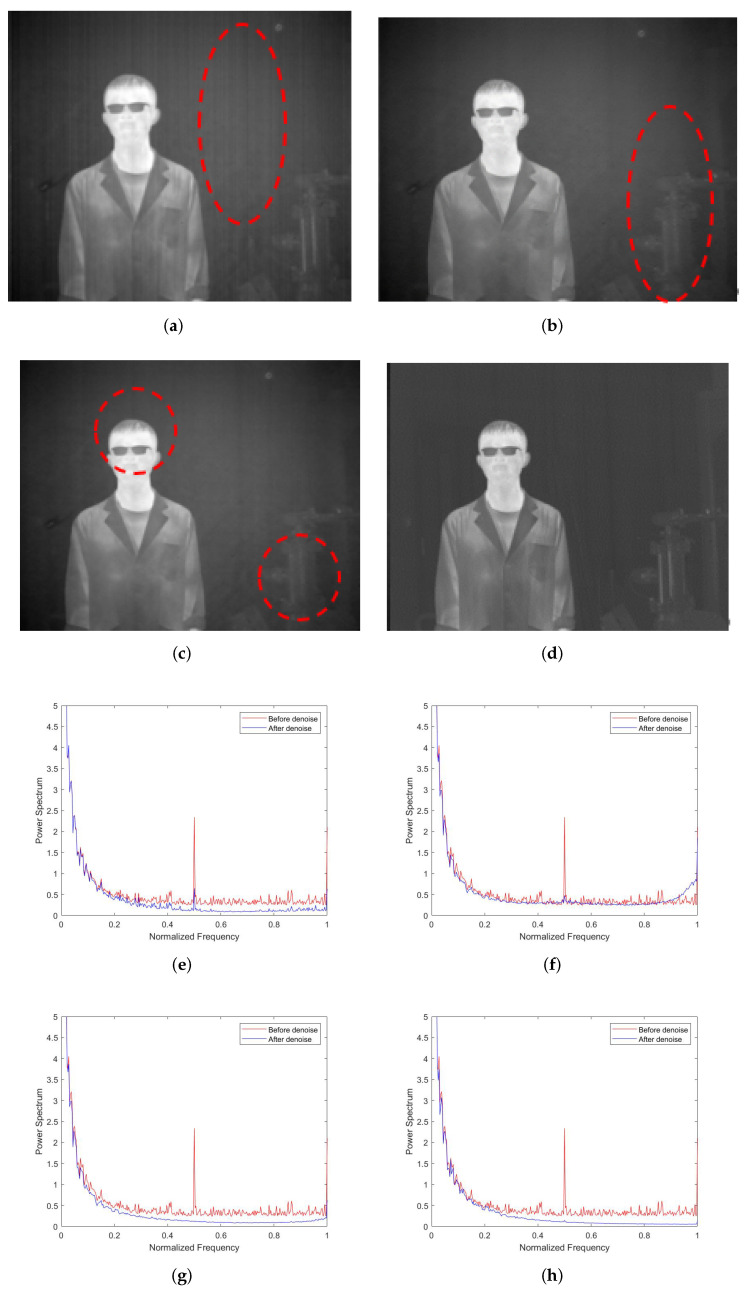
Denoising effects of different methods on an image of a person: (**a**) MSGF; (**b**) WAGE; (**c**) TVGF; (**d**) our approach; (**e**) MSGF; (**f**) WAGE; (**g**) TVGF; (**h**) our approach.

To verify the effectiveness of our approach, several experiments were performed on images containing stripe noises. The first experiment was reported in Figure 11. As shown in Figure 11a, the MSGF achieved a relatively poor denoising effect, and could not effectively identify and remove the irregular stripe noises with a small cross-directional gradient variation. As shown in Figure 11b, the WAGE method still leaves a small number of stripe noises unremoved. As shown in Figure 11c, the TVGF over-smoothed the original image in the horizontal direction. As a result, the edges of the person and the vertical features of the object were very obscure. As shown in Figure 11d, our approach effectively removed irregular stripe noises, while preserving the information of edge textures as much as possible. Figure 11e–h compare the mean power spectral densities (MPSDs) of all rows in the images denoised by different methods with the MPSD of all rows in the original image, where the abscissa is the normalized frequency, and the ordinate is the MPSD of all rows. Figure 11e,f clearly show small pulses at the locations of large pulses in the original image, indicating that a few stripe noises were not removed. Figure 11g,h performed well in MDSP, which is consistent with the performance shown in Figure 11a–d. The comparison fully demonstrates the superiority of our approach in the removal of stripe noises.

**Figure 12:**
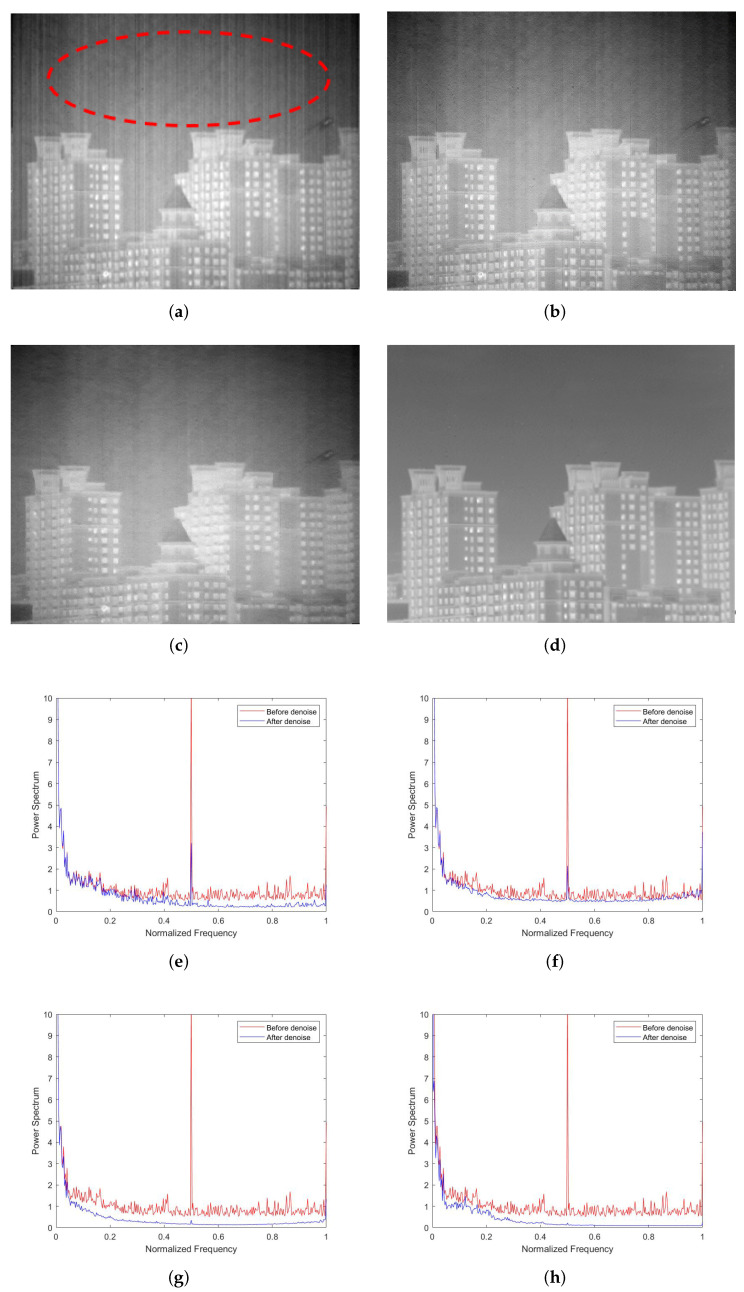
Denoising effects of different methods on an image of buildings against the sky: (**a**) MSGF; (**b**) WAGE; (**c**) TVGF; (**d**) our approach; (**e**) MSGF; (**f**) WAGE; (**g**) TVGF; (**h**) our approach.

The second experiment is reported in Figure 12. The original image is an infrared image containing several buildings against the sky. The objects in the image have a small gray difference from the background, making it difficult to differentiate between building edges and stripe noises through frequency domain filtering. As shown in Figure 12a,b, MSGF and the WAGE displayed poor denoising effects, despite preserving the edges, and left clear stripe noises in the background. As shown in Figure 12c, TVGF caused a loss of information due to over-smoothing of building edge pixels. As shown in Figure 12d, our approach eliminated stripe noises and retained the edges and details of the buildings. The MDSPs in Figure 12e–h were consistent with the performance of our approach. The above results further demonstrate the superiority of our approach.

**Figure 13:**
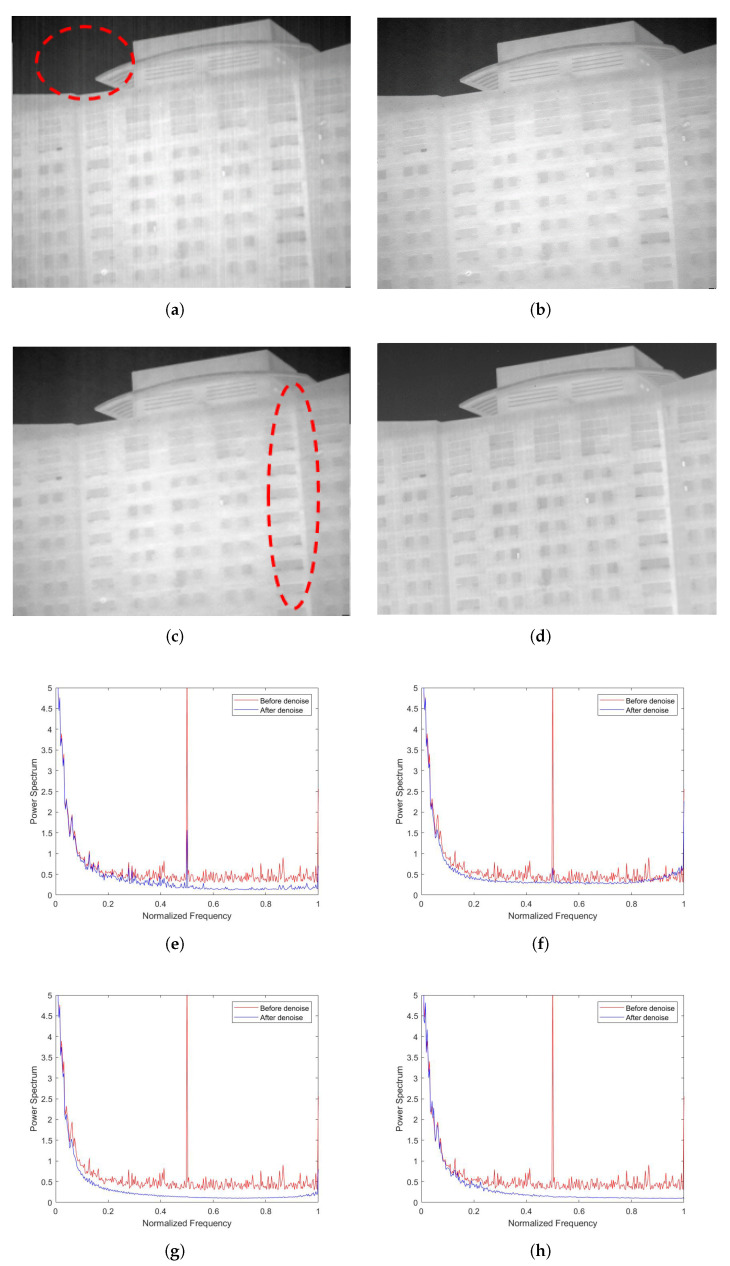
Denoising effects of different methods on an image of a single building: (**a**) MSGF; (**b**) WAGE; (**c**) TVGF; (**d**) our approach; (**e**) MSGF; (**f**) WAGE; (**g**) TVGF; (**h**) our approach.

The third experiment is reported in Figure 13. The original image depicts a single building with a vertical structure. As shown in Figure 13a,b, MSGF and the WAGE had the worst performance among all methods. The image edges and details were preserved, but the noises were not effectively erased. As shown in Figure 13c, TVGF led to clear attenuation of edge features, despite its good denoising effects. Additionally, as shown in Figure 13d, our approach preserves the edge information of the image and effectively achieves streak noise removal simultaneously. The MDSPs in Figure 13e–h were consistent with the performance of our approach. The above results provide more evidence for the superiority of our approach.

**Figure 14:**
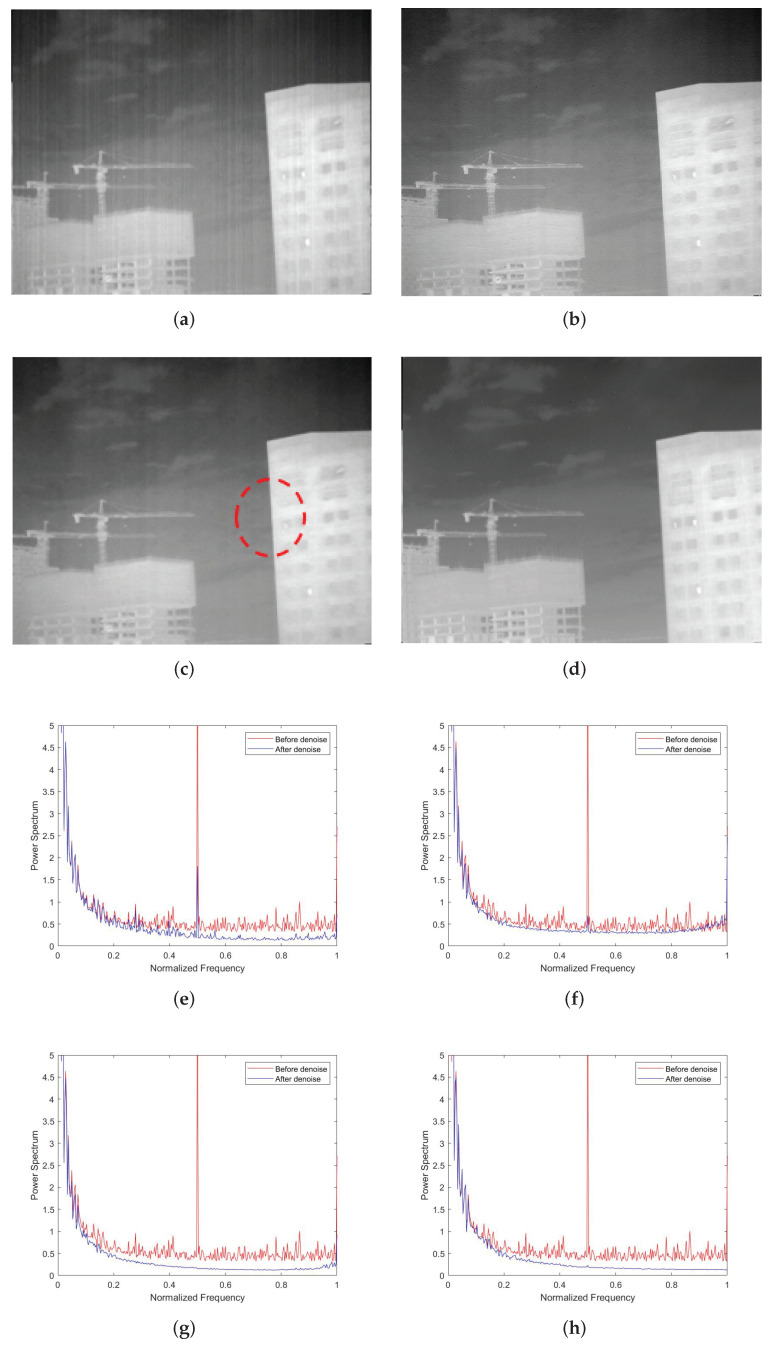
Denoising effects of different methods on an image of complex buildings: (**a**) MSGF; (**b**) WAGE; (**c**) TVGF; (**d**) our approach; (**e**) MSGF; (**f**) WAGE; (**g**) TVGF; (**h**) our approach.

The last experiment is reported in Figure 14. As shown in Figure 14a,b, MSGF and WAGE were outshined by the other methods, as they failed to remove some stripe noises. As shown in Figure 14c, the edges in images denoised by the TVGF method are blurred to varying degrees (encircled by red dotted lines). The MDSPs in Figure 14e–h were consistent with the performance of our approach. The above results fully reflect the superiority of our approach.

Table 1 compares the NR, MRD, and ID values of the images denoised by the four methods. The optimal value of each metric is shown in bold.

As shown in Table 1, our approach had the best effects in terms of the NR and MRD, which mirror the ability to denoise stripe images. Judging by the ID, our approach also achieved fairly good results. Some methods had a high ID as they were unable to fully remove noises and retained only part of the information of the original image. Through subjective observation of Figure 11, Figure 12, Figure 13 and Figure 14, the images denoised by our approach were not greatly distorted, had edge details preserved, and had ID values close to 1. These results testify that our approach is excellent in the removal of stripe noises.

## 5. Conclusions

This paper proposes a stripe noise removal model for infrared images, based on the sparse representation of L1-norm and the AEPO. The proposed model fully utilizes the directional, cross-directional, and structural differences between stripe noises in infrared images and other components in those images, and describes the sparsity of infrared images well with the L1-norm. Focusing on the edge pixels, the AEPO can reasonably separate and remove stripe noises, and can preserve the edge information of the original image excellently. The classic ADMM algorithm was introduced to solve the proposed model. Finally, the superiority of our approach was demonstrated through numerous experiments. Nevertheless, there are still some questions in the field of stripe noise removal. Our proposed method is flawed in dealing with diagonal stripe noises or heavy stripe noises; in the future, we will focus on removing these noises from infrared images.

## Figures and Tables

**Table 1:** Metrics of different methods on different images.

Image	Metric	MSGF	WAGE	TVGF	Non-AEPO	Our Approach
A person	NR	2.02	2.51	3.58	4.01	4.07
	MRD(%)	2.95	3.72	4.53	3.52	3.01
	ID	0.999	0.992	0.975	0.980	0.989
Buildings against the sky	NR	2.29	2.79	3.45	3.91	3.96
	MRD(%)	3.94	4.36	5.47	4.45	4.11
	ID	0.999	0.991	0.971	0.978	0.986
A single building	NR	3.31	3.36	3.41	3.48	3.53
	MRD(%)	2.74	2.56	3.40	2.96	2.47
	ID	0.999	0.993	0.977	0.985	0.991
Complex buildings	NR	3.05	3.16	3.40	3.36	3.42
	MRD(%)	3.08	2.47	4.10	3.54	2.21
	ID	0.999	0.993	0.980	0.988	0.994

## Data Availability

Due to the nature of this research, participants of this study did not agree for their data to be shared publicly, so supporting data is not available.
